# The antinutritional and vitamin composition of high‐quality yam flour as affected by yam specie, pretreatment, and drying method

**DOI:** 10.1002/fsn3.619

**Published:** 2018-10-02

**Authors:** Abdul‐Rasaq A. Adebowale, Abimbola B. Wahab, Philip O. Sobukola, Adewale O. Obadina, Ester O. Kajihausa, Olanike M. Adegunwa, Oladimeji L. Sanni, Keith Tomlins

**Affiliations:** ^1^ Department of Food Science and Technology Federal University of Agriculture Abeokuta Nigeria; ^2^ Department of Hospitality and Tourism Federal University of Agriculture Abeokuta Nigeria; ^3^ Natural Resources Institute University of Greenwich Greenwich UK

**Keywords:** antinutritional composition, *Dioscorea* sp., vitamin contents, Yam

## Abstract

Fresh yams are not shelf stable due to its high susceptibility to physiological deterioration; hence, its conversion into a more stable product like flour can stimulate its industrial application as a substitute to wheat flour. The influence of yam species, pretreatment, and drying method on the antinutritional factor and vitamin composition of high‐quality yam flour (HQYF) was determined. Four different yam species were pretreated with 0.28% potassium metabisulphite for 15 min and blanching at 70°C for 15 min. The differently pretreated slices were thereafter dried with cabinet dryer at 60°C for 48 h and open sun for 3 days, then milled into flour. The flour samples were analyzed for antinutritional and vitamin contents using standard laboratory procedures. The antinutritional factors in the high‐quality yam flour were significantly (*p* < .05) affected by yam specie, pretreatment, and drying methods. The low level of alkaloids (0.02 mg/100 g) and phytates (13.43 mg/100 g) in the flour samples from *D*. *rotundata* in this study underscores its safety for absorption in the body when consumed/used as food formulations. The main and interactive effect of specie of specie, pretreatment, and drying methods influenced the vitamin content of the high‐quality yam flour significantly (*p* < 0.05). The appreciable level of vitamin C (20.87–30.91 mg/100 g) recorded in all the HQYF could indicate the product of good nutritional quality for the consumers.

## 1 INTRODUCTION

Yam (*Dioscorea* spp.) tubers are one of the major staple crops of several people and communities in tropical regions especially in West Africa (Martine & Mario, 1991). Over 600 species of yam (out of which only few are cultivated for food) have been reported (IITA, 2006). The economically cultivated yam species are white yam (*Dioscorea rotundata poir*), water yam (*Dioscorea alata*), bitter yam (*Dioscorea dumetorum*), and yellow yam (*Dioscorea cayenesis*) (Aregahegn, Chandravanshi, & Atlabachew, 2013; McAnuff, Omoruyi, Sotelo‐López, & Asemota, 2005). Bhandari, Kasai, and Kawabata (2003) reported that there are several different edible yam species and available in different tropical regions, which differ in their chemical composition and nutritional importance. Many species and cultivars of edible yams are not consumed raw because of itchiness, bitterness, or toxicity component in the raw tuber. The alkaloid and saponin contents in yam may contribute to its bitterness or acute toxicity (Okwu & Ndu, 2006). So far the antinutrient compositions of the economically important species of yam have not been widely reported. Antinutritional factors when present in a food system lower the bioavailability of protein and minerals (Udensi, Oselebe, & Onuoha, 2010). Yams are very good sources of nutrients including carbohydrate, energy, vitamins (mostly vitamin C), proteins, and minerals. Some cultivated yam species have been reported to be rich in phosphorous and vitamins such as ascorbic acid, thiamine, niacin, and riboflavin (Dabonne, Kouakou, Martin, Hubert, & Kouame, 2011; Osunde, 2008).

Findings of Afoakwa and Sefa‐Dedeh (2001) and Afoakwa, Polycarp, Budu, Mensah‐Brown, and Otoo (2013) indicated that fresh yam tubers are perishable and are subject to deterioration during storage. Postharvest losses of yam tubers during storage are caused by physiological, biochemical, and microbial processes such as sprouting, transpiration, respiration, and rot as well as the activities of insects and nematodes (Hsu, Chen, & Tseng, 2003; Osunde, 2008). The feasibility of developing stable form of yam products for the health food markets has been reported (Chin‐Yin, Chin‐Lin, Wenlung, & Yih‐Ming, 2003; Wahab et al., 2016) due to the fact that yams are regarded as health foods and not staple foods in oriental countries (Hsu et al., 2003). As flours can be easily stored for long period of time and conveniently used in manufacturing formulated foods, therefore there is need to develop dried yam flour for diverse applications in food processing.

Drying is a traditional process commonly used to extend shelf life as well as enhancing the sensory qualities of many food products. Sun drying has been reported to generate 59%–71% of niacin losses in *D*. *schimperiana* yam (Afoakwa et al., 2013). Drying time and nutritional losses during drying of yam tubers could be reduced by the use of appropriate pretreatment and drying method/condition. Although sun drying is the common method of drying of yam in the tropics, it has a main disadvantage of slowness of the drying process and mold growth due to ambient temperature that is used for drying. There is therefore the need for more efficient drying methods that will produce a better quality product within a shorter drying period (Wahab et al., 2016). Product pretreatment has also been considered as a means of accelerating drying (Kudra, 2004; Wahab et al., 2016). Studies on local techniques of producing yam flour indicated that precooking is a crucial step but the resulting cooked products are usually characterized by a browning color as a result of enzymatic oxidation of polyphenols (Akissoe, Joseph, Christian, & Nago, 2003). Reduction in yam size as small thickness disks before blanching in hot water reduces the drying time, limits browning reaction, and improves the color of the resulting cooked products (Hounhouigan et al., 2000). By choosing suitable yam specie, pretreatment, and drying methods, the final flour quality can be controlled. This study aimed at determining the influence of yam species, pretreatment, and drying method on the antinutritional and vitamin composition of high‐quality yam flour.

## 2 MATERIALS AND METHODS

### 2.1 Raw material sourcing

Four freshly harvested and wholesome tubers of yam species namely *D. alata, D. rotundata*,* D*. *dumetorum, and D. cayenesis* obtained from local markets in Abeokuta, Ogun State, Nigeria were used for the study.

### 2.2 Processing of high‐quality yam flour

High‐quality yam flour was processed according to the procedures described by Wahab et al. (2016). The freshly harvested yam tubers were washed thoroughly to remove sand and other dirts. The washed tubers were then peeled separately using a stainless steel knife. The peeled tubers were sliced into 1 mm pieces using a stainless steel vegetable slicer. The slices were then washed in distilled water. The yam slices were divided into two equal portions; one portion was blanched in water bath at 70°C for 15 min, while the other portion was immersed in 0.28% potassium metabisulphite for 15 min. For cabinet drying, each pretreated yam slices were dried at 60°C for 24 h. For sun drying, each pretreated yam slices were dried on black polythene nylon for 2 days. The dried slices were milled separately with laboratory hammer mill and sieved using a 250 μm mesh to obtain yam flour herein referred to as high‐quality yam flour. The flour samples were packaged in airtight plastic container and stored at 4°C for further analysis.

### 2.3 Determination of antinutritional factors

The gravimetric determination of alkaloids followed procedure described by Harbone (1973). The method of Harbone (1973) was used to determine the total phenol content. The phytate was determined according to procedure of Wheeler and Ferrel (1979), the method of Swain (1979) was used for the determination of tannin contents of the differently processed yam flour. Oxalate was determined using methods described by Day and Underwood (1986). The Spectrophotometric method of Brunner (1984) was used for saponin analysis. AOAC (2000) procedures were used to determine vitamins B_1_, B_2_, and C, while the method of Guilarte, Shane, and Mcintrye (1981) was used to determine vitamin B_6_ content of the flour samples.

## 3 RESULTS AND DISCUSSION

### 3.1 Antinutritional compositions of HQYF samples

The results of the antinutritional content of the flour samples are presented in Table 1. The values ranged from 165–979 mg/100 g, 123–440 mg/100 g, 13.4–79.2 mg/100 g, 1.61–13.0 mg/100 g, 0.02–0.11 mg/100 g, and 9.02–49.3 mg/100 g for total phenol, tannin, phytate, saponin, alkaloid, and oxalate, respectively. The main and combined effects of specie, pretreatment, and drying methods on the antinutritional composition of the high‐quality yam flour (HQYF) were significant (*p* < 0.05) except for saponin in which pretreatment had no significant effect on. The presence of antinutritional factors may adversely affect the nutritive value of foods. Phenolic compounds have been reported to inhibit the activity of hydrolytic and digestive enzymes such as trypsin, amylase, lipase, and chymotrypsin (McAnuff et al., 2005). Phenols have been found to be responsible for bitterness and astringency associated with many foods (Bravo, 1998; McAnuff et al., 2005). It also exhibits an inhibitory effect on fungi in vitro (Choi, Jeong, & Lee, 2007; Ferraro, Piccirillo, Tomlins, & Pintado, 2016). Hence, the higher amount of phenols contained in bitter yam (*D. dumetorum)* flour in this study may contribute to its bitter taste and its resistance to fungal infection. The low level of phenol (164.5 mg/100 g) observed in *D*. *alata* flour was found to be within the range (0.16–0.25%) reported by Udensi et al. (2010) in the tubers of *D*. *alata* varieties. The presence of phenols has been suggested to indicate that *Dioscorea* species could act as anti‐inflammatory, antioxidants, anticlotting, immune enhancers, and hormone modulators (Okwu & Omodamiro, 2005).

**Table 1:** Antinutritional composition (mg/100 g) of HQYF as affected by species, pretreatments, and drying methods

Species	Pretreatments	Drying methods	Phenol	Tannin	Phytate	Saponin	Alkaloid	Oxalate
*D. rotundata*	Blanching	Cabinet	232	152	19.3	2.89	0.04	12.7
		Sun	180	132	19.2	3.62	0.02	9.02
	Potassium	Cabinet	472	122	31.6	5.95	0.03	16.9
		Sun	426	200	13.4	3.44	0.06	16.8
*D. dumetorum*	Blanching	Cabinet	573	220	66.6	6.14	0.08	20.2
		Sun	817	410	74.1	12.43	0.10	31.8
	Potassium	Cabinet	979	213	72.8	13.01	0.11	49.3
		Sun	873	440	61.9	9.36	0.11	21.6
*D. alata*	Blanching	Cabinet	209	132	79.2	3.57	0.06	10.7
		Sun	164	142	37.5	3.75	0.06	11.0
	Potassium	Cabinet	574	242	33.3	3.41	0.11	25.3
		Sun	553	230	38.3	2.16	0.10	23.0
*D. cayenesis*	Blanching	Cabinet	226	185	28.5	5.75	0.04	12.0
		Sun	513	149	51.4	3.65	0.09	21.4
	Potassium	Cabinet	258	198	14.7	4.00	0.07	15.1
		Sun	421	218	15.5	1.61	0.09	15.8
Range			164–979	122–440	13.4–79.2	1.61–13.0	0.02–0.11	9.02–49.3
Mean			467	212	41.1	5.29	0.07	19.5
SD			255	91.9	23.3	3.44	0.03	10.0
SE			64.0	23.0	5.82	0.86	0.01	2.5
*p* of specie (S)			a	a	a	a	a	a
*p* of pretreatment (P)			a	a	a	ns	a	a
*p* of drying method (D)			a	a	a	a	a	a
*p* of S x P			a	a	a	a	a	a
*p* of S x D			a	a	a	a	a	a
*p* of P x D			a	a	a	a	ns	a
*p* of S x P x D			a	a	a	a	a	a

a Significant at *p* < 0.05 and ns not significant at *p* > 0.05.

Afiukwa et al. (2013) reported that protein digestibility and palatability are reduced when tannin forms complexes with protein. However, their contents in foods are known to reduce through cooking (Lewu, Adebola, & Afolayan, 2010). Tannin concentration in the flour samples was relatively higher when compared with the earlier values reported for *D. rotundata* (20 mg/100 g) by Uka (1985), *D. alata* (46.5–180.3 mg/100 g) by Udensi et al. (2010), and 20–255 mg/100 g reported on various underutilized *Dioscorea* tubers by Arinathan, Mohan, and Maruthupandian (2009). The bitter characteristics of *D. dumetorum* may be due to the high level of tannin found in it. The trace quantities of tannin available in yam tubers act as a repellent against rot in yam (Okwu & Ndu, 2006). Phytates and oxalates have been reported to adversely affect the bioavailability of mineral in humans (Bhandari & Kawabata, 2006). The value of phytic acid obtained in the present study is low compared with 58.6–198 mg/100 g obtained for cultivars of *D*. *alata* by Wanasundera and Ravindran (1994). Oxalate levels were also very low (9.02–49.3 mg/100 g) compared with 483–781 mg/100 g reported by Wanasundera and Ravindran (1994) but higher than 0.20–0.63 mg/100 g reported by Polycarp, Afoakwa, Budu, and Otoo (2012).

The availability of alkaloids in the tubers of *Dioscorea species* indicates that yam tubers should not be eaten raw. The level of alkaloid (0.02–0.11 mg/100 g) in this study was lower compared with that reported for different yam varieties by Okwu and Ndu (2006). The high content (0.11 mg/100 g) of alkaloids in *D*. *dumetorum* lends credibility to the reports of toxicities associated with its use (Afiukwa et al., 2013; Eka, 1998). However, the low level of alkaloid found in D. alata is within the range of 0.12%–0.55% reported by Udensi et al. (2010). This underscores the safety of the *D*. *alata* specie when consumed, as most alkaloids are known to be toxic and can cause a wide range of physiological changes in the body when consumed (Awa & Chinedum, 2015). However, simple processing such as cooking removes the alkaloids present in most cultivated species of yams (Cemaluk, Daniel, & Nkiru, 2014).

Saponins and alkaloids are considered important due to their toxicity in yams (Okwu & Ndu, 2006). This toxic metabolite occurs in varying concentration in yam tubers. The saponin content of the yam flour was lower than 2.98–19.5 mg/100 g reported by Okwu and Ndu (2006). The high level (13.0 mg/100 g) of saponin found in *D*.* dumetorum* sample may be responsible for its characteristic bitter taste. Saponins natural tendency to ward off microbes makes them good candidates for treating fungal infections (Okwu & Ndu, 2006). These compounds have been reported to serve as natural antibiotics, which help the body to fight infections and microbial invasion (Sodipo, Akiniyi, & Ogunbanosu, 2000). The antinutritional factors of the flour samples were significantly affected by pretreatment and drying methods. However, the antinutritional content of samples pretreated with blanching was generally lower than those treated with potassium metabisulphite.

### 3.2 Vitamin contents of HQYF samples

The vitamin contents of the HQYF samples ranged from 0.18–1.05 mg/100 g (vitamin B_1_), 0.44–4.55 mg/100 g (vitamin B_2_), 2.00–4.34 mg/100 g (vitamin B_6_), and 20.8–30.9 mg/100 g (vitamin C) as shown in Table 2. The main effect of species and the interactive effects of species, pretreatment, and drying method significantly (*p* < 0.05) affected the vitamin contents of the yam flour samples. However, the combined effect of pretreatment and drying method had no significant (*p* > 0.05) effect on all the vitamins determined except for vitamin B_6_. Whereas, the interactive effect of species and drying methods was significant (*p* < 0.05) on all vitamins determined. Generally, samples treated with potassium metabisulphite had higher vitamin B contents, while the vitamin C contents of flour from blanching pretreatment were higher. The sun‐dried samples, irrespective of variety and pretreatment, recorded the lowest ascorbic acid content. This corroborates the findings of Gallali, Abujnah, and Bannani (2000) who reported appreciable loss of ascorbic acid content in sun‐dried tomato powder than the oven and solar‐dried samples. The vitamin C level (20.9–30.9 mg/100 g) reported in this work was higher than the value recorded for plantain flour 6.30 mg/100 g (Adetuyi & Komolafe, 2011) and traditional yam flour (*elubo*) by Jonathan, Ajayi, and Omitade (2011), but lower than the value obtained from flour of different varieties of water yam (16.7–35.2 mg/100 g) by Udensi et al., (2008). Ascorbic acid activates the functions of the body cells, and it is a powerful antioxidant. Vitamin C enhances the absorption of iron in the intestine, fight against infections, neutralizes blood toxins, and intervenes in the healing of wounds (Roger, 1999). According to Afiukwa et al. (2013), the mean ranges of thiamine and vitamin C in cultivated Nigerian yams are 0.01–0.11 and 4.00–18.0 mg/100 g, respectively. The lower values obtained in this study may be as a result of genetic and interspecies variations, cultural practices, and environmental factors. Thiamine (vitamin B_1_) functions as a coenzyme in the phosphogluconate pathway. Thiamine is needed for proper digestion.

**Table 2:** Vitamin contents (mg/100 g) of HQYF as affected by species, pretreatments, and drying methods

Specie	Pretreatments	Drying methods	Vitamin B_1_	Vitamin B_2_	Vitamin B_6_	Vitamin C
*D. rotundata*	Blanching	Cabinet	0.29	1.39	2.27	29.1
		Sun	0.29	4.55	3.56	27.0
	Potassium	Cabinet	0.36	1.16	2.08	25.8
		Sun	0.18	0.97	2.00	27.1
*D. dumetorum*	Blanching	Cabinet	0.93	1.20	3.36	24.0
		Sun	0.37	1.06	4.34	22.2
	Potassium	Cabinet	1.05	0.46	3.95	21.8
		Sun	0.95	3.48	3.70	23.2
*D. alata*	Blanching	Cabinet	0.47	1.39	2.36	25.2
		Sun	0.57	0.44	2.92	20.9
	Potassium	Cabinet	0.36	1.19	2.73	24.1
		Sun	0.39	1.75	2.40	21.4
*D. cayenesis*	Blanching	Cabinet	0.31	2.36	2.09	23.0
		Sun	0.60	1.77	2.03	30.9
	Potassium	Cabinet	0.41	3.52	2.96	28.8
		Sun	0.37	2.57	2.44	24.6
Range			0.18–1.05	0.44–4.55	2.00–4.34	20.9–30.91
Mean			0.49	1.83	2.82	24.93
SD			0.25	1.13	0.73	2.87
SE			0.06	0.28	0.18	0.72
*p* of specie (S)			a	a	a	a
*p* of pretreatment (P)			a	ns	ns	a
*p* of drying method (D)			a	a	a	ns
*p* of S x P			a	a	a	ns
*p* of S x D			a	a	a	a
*p* of P x D			ns	ns	a	ns
*p* of S x P x D			a	a	a	a

a Significant at *p* < 0.05 and ns not significant at *p* > 0.05.

## 4 CONCLUSIONS

Species, pretreatment, and drying methods had a significant effect on most of the antinutritional and vitamin contents of the yam flour. The antinutritional factors were significantly reduced by blanching compared with the use of potassium metabisulphite as the method of pretreatment.
